# Dietary fiber, starch, and sugars in bananas at different stages of ripeness in the retail market

**DOI:** 10.1371/journal.pone.0253366

**Published:** 2021-07-08

**Authors:** Katherine M. Phillips, Ryan C. McGinty, Garret Couture, Pamela R. Pehrsson, Kyle McKillop, Naomi K. Fukagawa

**Affiliations:** 1 Department of Biochemistry, Virginia Tech, Blacksburg, Virginia, United States of America; 2 University of California Davis, Davis, California, United States of America; 3 United States Department of Agriculture, Agricultural Research Service, Beltsville Human Nutrition Research Center, Beltsville, Maryland, United States of America; Embrapa Agroindústria Tropical, BRAZIL

## Abstract

The goal of this work was to evaluate changes in dietary fiber measured by the traditional enzymatic-gravimetric method (AOAC 991.43) and the more recently accepted modified enzymatic-gravimetric method (AOAC 2011.25), mono- and disaccharides, and starch as a function of assessed ripeness in a controlled study of a single lot of bananas and in bananas at the same assessed stages of ripeness from bananas purchased in retail stores, from different suppliers. Sugars, starch, and dietary fiber were analyzed in bananas from a single lot, at different stages of ripeness, and in retail samples at the same assessed stages of ripeness. Mean fiber measured by the traditional enzymatic-gravimetric method (EG) was ~2 g/100g and not affected by ripeness. Mean fiber assessed with the recently modified method (mEG) was ~18 g/100g in unripe fruit and decreased to 4–5 g/100g in ripe and ~2 g/100g in overripe bananas. Slightly ripe and ripe bananas differed by ~1.1 g/100g in the controlled single-lot study but not among retail samples. There was a large increase in fructose, glucose and total sugar going from unripe to ripe with no differences between ripe and overripe. Aside from stage of ripeness, the carbohydrate composition in retail bananas is likely affected by differences in cultivar and post-harvest handling. Results from this study demonstrate the importance of measuring dietary fiber using the mEG approach, developing more comprehensive and sensitive carbohydrate analytical protocols and food composition data, and recognizing the impact of different stages of maturity and ripeness on carbohydrate intake estimated from food composition data.

## Introduction

Bananas are one of the most widely consumed fruits according to recent estimates [1,2] and rank fourth after rice, wheat and corn among the world’s most economically important food crops [3]. The fruit is a dietary staple for hundreds of millions of people across Asian, African and American tropics and are an economic source of nutritious calories (Heslop-Harrison & Schwarzacher, 2007) [5]. In the past century, the medical and health communities have defended the nutritional value of the banana when its digestibility was in question [4]. There are over a thousand domesticated *Musa* cultivars characterized by significant genetic diversity, with challenges to banana production from virulent diseases, abiotic stresses, and demands for sustainability, quality, transport, and yield [5]. Accordingly, understanding factors that impact nutrient profiles is important as new technologies are employed to solidify the position of the banana as a staple food and cash crop. The most commonly consumed cultivar in the U.S. is *Musa acuminate*, which includes the Cavendish, distinguished by the plant height and familiar features of the fruits [6].

Carbohydrates are major components of the banana fruit and comprise approximately 20% of the fruit on an as-consumed basis [[7], NDB# 009040], equivalent to ~80% of the dry weight. The primary carbohydrates are starch, sugars (fructose, glucose, sucrose), and non-starch polysaccharides (for example, pectin, cellulose, hemicellulose) that are part of “dietary fiber” [8]. Currently, the U.S. Food and Drug Administration (FDA) defines dietary fiber as the “nondigestible soluble and insoluble carbohydrates (with 3 or more monomeric units), and lignin that are intrinsic and intact in plants; isolated or synthetic non-digestible carbohydrates (with 3 or more monomeric units) determined by FDA to have physiological effects that are beneficial to human health.” [9]. This definition codifies what can be considered dietary fiber declared on food labels and includes components not measured by some methods. It also expands an earlier definition of dietary fiber to include “resistant starch,” that is, starch that is inaccessible to digestive enzymes due to native structure or retrogradation [10]. The starch content of bananas (*Musa acuminata*, “Cavendish”) has been reported to change from approximately 21 g/100g in unripe fruit to approximately 1 g/100g in fully ripe fruit [11]. During ripening there is a decrease in enzyme-resistant starch [12] and an increase in water-soluble pectin [13].

Food composition databases report average nutrient concentrations in foods, primarily for the purpose of estimating dietary intake. When these databases are compiled entries may or may not include the distinction of variables such as ripeness or maturity of fruits and vegetables. For example, the USDA Standard Reference database (SR Legacy, [7]) reports data for “bananas, raw”, with no distinction among bananas of different ripeness. A primary change during banana ripening is the breakdown of starch, including some types of resistant starch. However, this change in resistant starch content is not captured by traditional EG measurements. It is therefore reasonable to expect that the dietary fiber content and composition as measured by the newer mEG method would be influenced by the degree of ripening in bananas. Whereas controlled ripening or field research studies have reported on the fiber, starch and sugars in ripening bananas, there is also a need to understand whether any differences associated with different ripeness persist in a nutritionally significant amount, after all variables between production and point of sale as a “banana” in the retail market become involved.

The goal of this work was to evaluate changes in dietary fiber measured by the traditional enzymatic-gravimetric method [14] and the more recently accepted modified enzymatic-gravimetric method [15,16], mono- and disaccharides, and starch as a function of assessed ripeness. We report a controlled experiment of a single lot of unripe bananas assayed while unripe and at stages through overripe, as well as a study of bananas purchased from several retail stores, coming from different suppliers, at the same assessed levels of ripeness.

## Materials and methods

### Study design

For the ripening study, the carbohydrate content and composition of bananas (Cavendish) from a single lot of unripe bananas was evaluated at the unripe, slightly ripe, ripe, and overripe stages. Sugars (fructose, glucose, sucrose, maltose), starch, and dietary fiber were measured by the traditional enzymatic-gravimetric (EG) method (AOAC 991.43) and the newer modified EG (“McCleary”) method (AOAC 2011.25). Medium sized bananas were selected for this comparison. The effect of size was assessed at the unripe and ripe stages by additionally preparing samples of “extra-large” size bananas. Ripeness and size were defined according to specific and consistent criteria. Three samples, each comprising three to five bananas, were prepared for each ripeness/size. The inclusion of multiple bananas was intended to mitigate any variation in the precision of assessed ripeness, and also to provide enough material for analysis of different components. Additionally, samples of medium size ripe bananas were dehydrated and analyzed to gain preliminary data on the effect of dehydration on the dietary fiber content.

For the retail study, bananas (Cavendish) were sampled from different local retail markets, without regard to supplier or size, at the slightly ripe, ripe, and overripe stages. Since the goal of this study was to evaluate bananas as purchased and consumed and unripe bananas are not generally presented in retail markets, they were not sampled. Ripeness was assessed according to the same criteria used in the controlled ripening study. These samples were either purchased at the targeted degree of ripeness, or in some cases of slightly ripe or ripe samples some of the bananas were also allowed to ripen and also analyzed at a later stage of ripeness. Thus, the marketplace sampling involved unknown and uncontrolled variables such as producer, country of origin, supply chain, post-harvest handling, ripeness at point of purchase, fruit size. The results from the retail study were then compared to see if the conclusions relating carbohydrate composition and ripeness in the controlled experiment were evident among the marketplace samples.

### Sample procurement and preparation

For the ripening study, three cases of unripe bananas (Cavendish, stage 1 or 2 ripeness), each containing approximately ~18 kg (40 lb.) and comprising approximately 100 bananas, from the same brand, supplier, and lot were ordered in mid-July 2019 through a local retail outlet (Blacksburg, VA). After separating the individual bananas from each hand, they were segregated by size as previously defined and positioned in a single layer over a ~1.8 m^2^ (~3.0 m x 0.6 m) countertop with adequate air flow over and around each banana. After removing samples of unripe bananas to be prepared (as described below), the remaining bananas were allowed to ripen at ambient conditions; room temperature was controlled (19–22°C), however, (relative) humidity was uncontrolled (53–77%). At each targeted stage of ripeness, three samples each comprising three (for medium-size samples) or five (for extra-large samples) bananas were prepared for analysis (except the two medium-sized ripe bananas samples that were to be dehydrated, which each contained 7–12 bananas).

Once the ripeness and sizes of the individual unpeeled bananas had been assessed (as described below), they were peeled, one at a time, and size measurements of the peeled fruits were quickly taken. The fruit was then cut with a stainless steel paring knife into ~1-cm thick slices, immediately frozen in liquid nitrogen, and homogenized in liquid nitrogen using a 6-L Robot Coupe Blixer food processor (Robot Coupe USA. Jackson, MS) as described elsewhere [17], with subsamples (10–15 g each) for analysis of sugars, starch, dietary fiber, and moisture dispensed among 60-mL glass jars with Teflon™ lined lids (Qorpak. Clinton, PA) and immediately frozen at -60°C. The dehydrated samples were prepared by cutting each fruit into ~1 cm slices, then dehydrating in a single layer using a food dehydrator (Nesco Professional Food Jerky Dehydrator, Model# FD-75PR. Metalware Corp., Two Rivers, WI), run for 17 hours at 57°C, to approximately 25% of the original mass. The dried bananas were transferred to an aluminum foil-lined cookie sheet in a single layer, covered with cheesecloth, and allowed to sit at ambient conditions (the same as described for ripening) for 24 hours, and were then homogenized as described for the undried bananas.

**Fig 1:**
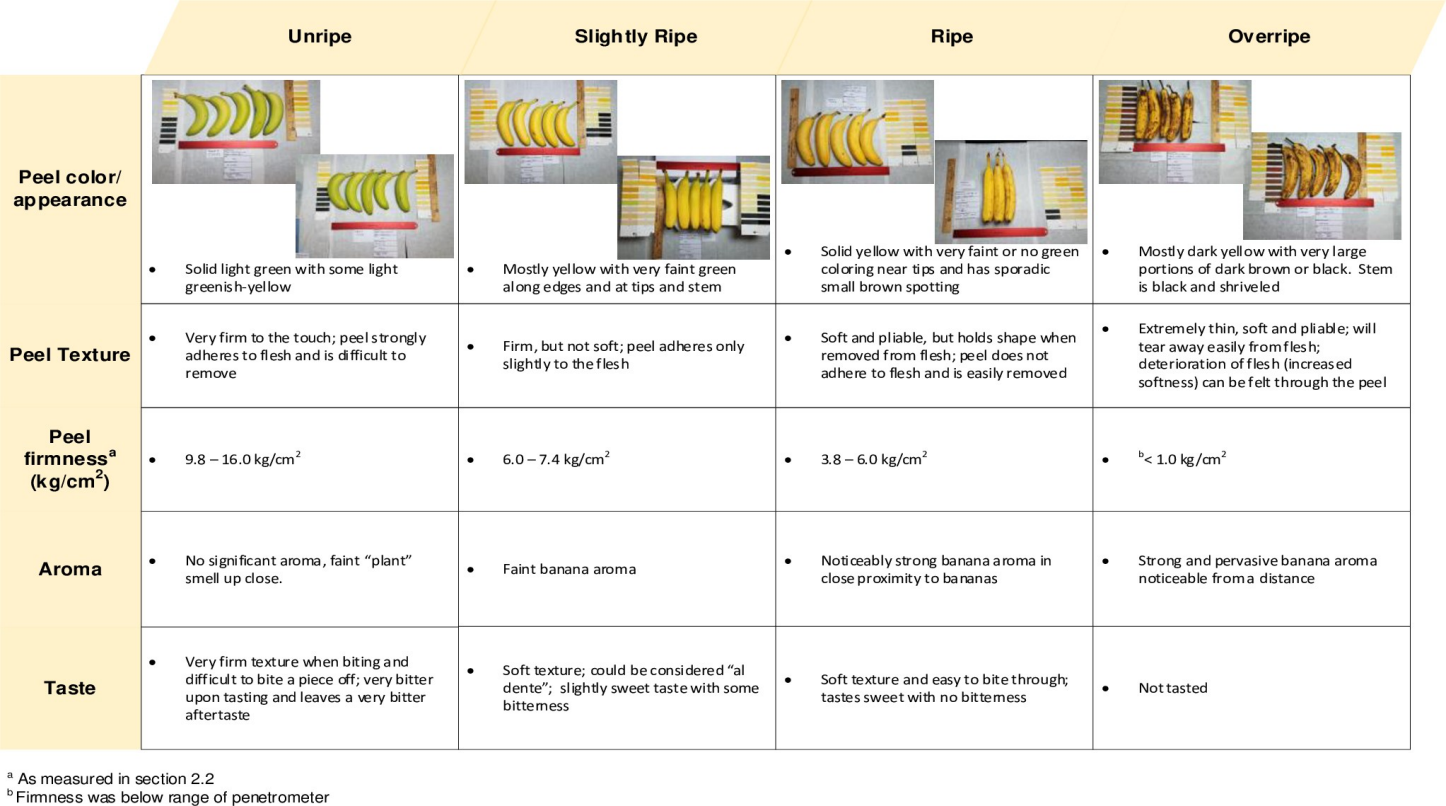
Criteria for assessed ripeness of bananas.

For the retail study, slightly ripe, ripe, or overripe bananas were sampled directly from local retail outlets (Blacksburg, VA), during the first two weeks of December 2019, from multiple brands and countries of origin. A total of six samples at each stage of ripeness were prepared, with ripeness assessed in the same manner as in the ripening study (see next section and Fig 1). Each sample comprised 1300–1500 g unpeeled weight. In some cases, a larger amount of slightly ripe or ripe bananas was purchased, with a portion of the sample allowed to ripen (as described above, except relative humidity was 27–50%) to yield a sample at a subsequent stage of ripeness. Banana size was not a criterion for the retail study, and each prepared sample included 5–12 bananas (~750–1200 g peeled weight).

### Determination of ripeness and size

The level of ripeness and size were assessed as summarized in Fig 1, and were consistent with USDA market inspection criteria [18] of stage 2 for “unripe”, 4–5 for “slightly ripe”, 6–7 for “ripe”, and 7+ for “overripe”. Measurements and visual and sensory evaluation were performed as follows. Peel color was matched to a standard color card deck (Aerospace Material Specification Standard 595A Color Fan Deck. AMS-STD-595) and documented by photographing the bananas with the matching color card(s) in frame, under standardized lighting. Sensory evaluation was performed by at least two persons who had the same independent assessment of a given fruit. Aroma was assessed by smelling the actual bananas (unpeeled) included in each prepared sample. Taste was done by destructive assessment of a banana(s) that was representative of the ripeness of the fruits being included in the prepared sample. Flesh firmness was measured using a manual penetrometer with ±0.1 kg/cm^2^ precision (Fruit Hardness Tester, Venus Instruments, VTSYIQIModel# GY-3), just before the peeled banana was sliced and homogenized (below), by inserting the 8-mm probe to a depth of 10 mm at each of three places on the fruit (~2.5 cm distal from stem end, midpoint, and ~3.5 cm from the tip).

Banana size was defined by length measurement of the unpeeled banana along the outer curve from tip to base of stem, measured with a flexible measuring tape, with “medium” being 21.6–23.5 cm (8.5–9.25 in.) and “extra-large” 28.6–30.5 cm (11.25–12.0 in.). The weight of each banana, unpeeled and peeled, was measured to the nearest 0.1 g, with the “nub” at the base of the banana and any fibrous phloem bundles being removed and included in the peel weight. For ripe bananas, if a peeled fruit had significantly bruised flesh it was excluded and replaced with another banana. Overripe, slightly ripe, and unripe banana flesh was used as is.

### Analytical methods

The sugar, starch, dietary fiber, protein, total fat, and ash assays were performed at certified commercial laboratories using standard methods of analysis. Dietary fiber was determined by two standard enzymatic-gravimetric methods, the traditional EG method (AOAC 991.43) [14] and the more recently developed enzymatic-gravimetric-liquid chromatography approach (AOAC 2011.25) [16] (mEG), without freeze-drying samples. The frozen (-60°C) subsamples, batched with control samples (see *Quality Control*), were shipped on dry ice via overnight delivery to the analytical laboratories (Eurofins Food Integrity & Innovation, Madison WI for mEG fiber and Merieux NutriSciences U.S., Crete IL for starch, sugars, EG fiber, and proximates other than moisture), and stored frozen until analyzed. Dietary fiber was determined by two standard enzymatic-gravimetric methods, the traditional EG method (AOAC 991.43) [14] and the more recently developed enzymatic-gravimetric-liquid chromatography approach (AOAC 2011.25) [16] (mEG), without freeze-drying samples. Sugars (mono-and disaccharides: fructose, glucose, sucrose, maltose) were assayed by liquid chromatography after water extraction of the homogenized samples (AOAC 980.13) [19]. Starch was measured by the enzymatic method, involving incubation of the autoclaved sample with amyloglucosidase and spectrophotometric measurement of released glucose (AOAC 979.10) [20], with glucose determined by glucose oxidase assay (Sigma Diagnostics Glucose Procedure No. 510. Sigma-Aldrich, St. Louis, MO). Protein was determined as Kjeldahl nitrogen*6.25 [21], ash by incineration [22], and total fat by acid hydrolysis [23]. Moisture was measured by vacuum drying 2-gram portions of the prepared samples at 65–70°C at 84.7 kPa to a constant weight (AOAC 934.01 using 65–70°C procedure) [24].

### Quality control

The banana samples were batched with control and/or commercially available certified reference materials for each nutrient analysis. The control materials (CC) had established tolerance limits based on ongoing use in USDA food composition studies [25]. A mixed starchy vegetable composite (“Starchy Vegetable II CC”) (canned spinach, potatoes without skin (drained), vegetarian vegetable soup without noodles, fat-free refried beans; baby food sweet potatoes and corn blend, and non-iodized salt) was analyzed for sugars and starch. A mixed vegetable homogenate (“Fiber Vegetable CC”) (canned chickpeas, raw orange-flesh sweet potatoes without skin, ripe bananas (peeled), baked russet potatoes without skin, raw kale leaves, and raw white onions without skin) was analyzed for fiber. Reference materials (RM) were procured from the National Institute of Standards and Technology (NIST) (Gaithersburg, MD) and included SRM® 3233 Breakfast Cereal for sugars and dietary fiber and NIST SRM® 2383a Baby Food for sugars.

Results for the CC and RM were evaluated relative to the expected tolerance limits for the CCs and the range from the certificate of analysis as well as the z-score for the assayed mean, calculated according to Engman et al. [26]. Additionally, select samples were analyzed in duplicate in each analytical batch to assess precision, using the HorRat ratio for the assayed relative standard deviation divided by the expected standard deviation [27]. Samples at the different stages of ripeness were also distributed evenly among analytical batches, to avoid conflating any systematic run to run analytical variability to samples.

### Data analysis

Means, standard deviations, percent relative standard deviation (RSD) and other calculations were done using Microsoft Excel (Microsoft Corp., Redmond, WA). Analysis of variance (ANOVA) and Tukey’s test with 95% confidence interval were used for means comparisons [28] using XLSTAT [29]. For comparison of nutrient concentrations in the dried and undried bananas, the assayed moisture content was used to calculate nutrient concentrations on a dry mass basis. To test for analytical differences between the ripening study samples and the retail samples, which were assayed several months apart, the mean values for control samples analyzed with samples at each time point and with 2 or more replicates at each time point were compared to test for statistical significance. These data included mEG in NIST SRM® 3233 and mEG, starch, and sugars in the Starchy Vegetable II CC.

## Results

### Quality control

**Fig 2:**
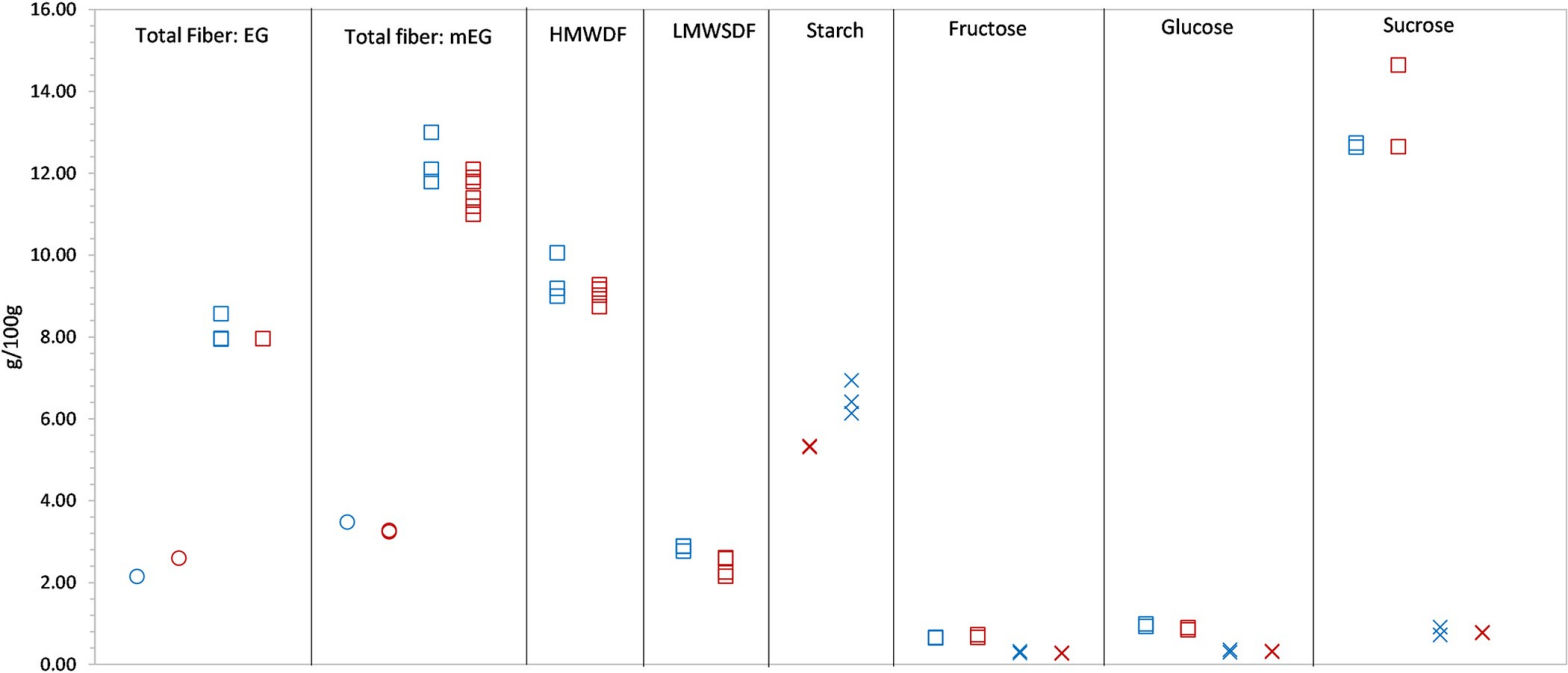
**Results for control materials (CC) analyzed with banana samples from the ripening study (blue) and the retail study (red).** EG = enzymatic-gravimetric method (AOAC 991.43) [14]; mEG = modified enzymatic-gravimetric method [16]; HMWDF = high molecular weight dietary fiber (insoluble dietary fiber and soluble dietary fiber precipitate) and LMWSDF = low molecular weight soluble dietary fiber (AOAC 2011.25) [16]. Circle (○) = Fiber Vegetable CC, Square (□) = NIST SRM®3233 Breakfast Cereal, X = Starchy Vegetable II CC (see Materials and Methods); reference ranges (target ± uncertainty, g/100g) for NIST SRM® 3233 [30]: EG total fiber, 7.8–10.2; mEG total fiber, 9.2–14.6; HMWDF, 6.4–12.0; LMWSDF, 1.8–4.2; fructose, 0.42–1.20; glucose, 0.68–1.40; sucrose, 12.67–14.17; maltose 0.37–0.55. Assayed maltose was <0.15 g/100g in all banana samples.

Results for the commercially available reference materials and established in-house control composites support the accuracy of the data (Table 1). Data for the control samples assayed with each batch of samples (Fig 2) illustrate the overall uncertainty across the separation in time of the analyses performed for the ripening study and retail study banana samples and multiple analytical batches for each component. HorRat values ranged from 2.3–7.6. Although higher than the expected ratio of < |3.0|, the HorRat for fiber components and for sugars with concentration < 1 g/100g were considered reasonable for the multi-step gravimetric assay and for the magnitude of variability that would be nutritionally meaningful. This contribution of potential analytical variability can be taken into account when considering the significance of differences between samples/treatments. Based on these data, analytical uncertainty between the analytical batches does not indicate differences that would bias comparisons among samples and ripeness as a result of analytical variability.

**Table 1:** Summary of results for commercial reference materials and control materials analyzed with samples from ripening study and retail study samples.

		Certificate of analysis^a^			Assayed				
Component	Material	Target ± Uncertainty			Mean	Range	%RSD	HorRat	Z-score^b^
**Total Dietary Fiber, EG**									
	Fiber Vegetable CC	n/a			2.38	0.45	13.4	7.6	
	NIST SRM® 3233 Breakfast Cereal	***7*.*8***	***-***	***10*.*2***	8.11	0.62	3.8	2.6	-6.9
**Total Dietary Fiber, mEG**									
	Fiber Vegetable CC	n/a			3.33	0.24	3.9	2.3	
	NIST SRM® 3233 Breakfast Cereal	***9*.*2***	***-***	***14*.*6***	11.8	2.00	5.0	3.6	-0.8
**High Molecular Weight Dietary Fiber (mEG)**									
	NIST SRM® 3233 Breakfast Cereal	***1*.*1***	***-***	***4*.*1***	9.21	1.32	4.5	3.1	0.1
**Low Molecular Weight Soluble Dietary Fiber (mEG)**									
	NIST SRM® 3233 Breakfast Cereal	***1*.*8***	***-***	***4*.*2***	2.60	0.73	11.2	6.5	-10.4
**Starch**									
	Starchy Vegetable II CC	n/a			6.03	1.63	11.7	7.7	
**Fructose**									
	NIST SRM® 3233 Breakfast Cereal	***0*.*42***	***-***	***1*.*2***	0.68	0.08	5.5	2.6	-8.1
	Starchy Vegetable II CC	n/a			0.29	0.05	7.4	3.1	
	NIST SRM® 2383a Baby Food	***3*.*87***	***-***	***4*.*05***	4.51	*		*	4.3
**Glucose**									
	NIST SRM® 3233 Breakfast Cereal	***0*.*68***	***-***	***1*.*4***	0.92	0.14	6.4	3.1	-5.9
	Starchy Vegetable II CC	n/a			0.33	0.06	8.1	3.4	
	NIST SRM® 2383a Baby Food	***3*.*69***		***3*.*91***	4.05	*		*	2.0
**Sucrose**									
	NIST SRM® 3233 Breakfast Cereal	***12*.*67***	***-***	***14*.*17***	13.17	2.01	7.5	5.5	-1.4
	Starchy Vegetable II CC	n/a			0.80	0.19	10.1	4.9	
	NIST SRM® 2383a Baby Food	***3*.*45***		***3*.*69***	3.40	*		*	-1.4

*n = 1, so no HorRat or range could be calculated.

^a^For commercial reference materials, reference range from certificates of analysis [30,31].

“HorRat” is the ratio of the assayed relative standard deviation (RSD) to the expected standard deviation based on analyte concentration [27]. EG = enzymatic-gravimetric method (AOAC 991.43) [14]; mEG = modified enzymatic-gravimetric method, with high molecular weight dietary fiber comprising insoluble dietary fiber and soluble dietary fiber precipitate and low molecular weight soluble dietary fiber (AOAC 2011.25) [16]. (see Materials and Methods for description of control materials). Assayed maltose was <0.5 g/100g in NIST SRM® 3233 Breakfast Cereal (reference mean ± uncertainty = 0.37–0.55 g/100g [30]).

### Carbohydrate content and composition of bananas at different ripeness

#### Ripening study

**Fig 3:**
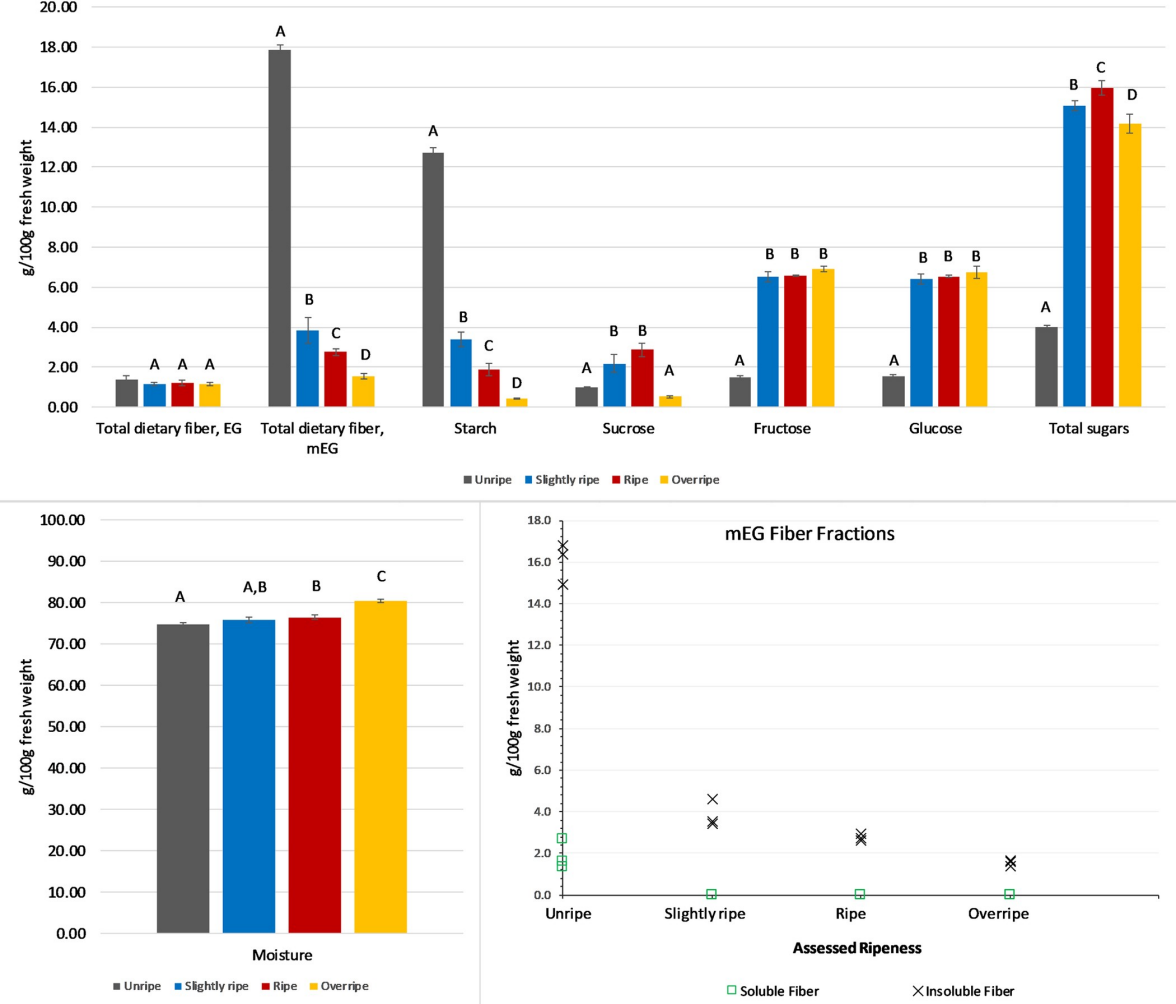
Changes in content of sugars, starch, and dietary fiber in a single lot of bananas assayed at different stages of ripeness. Values shown are the mean ± standard deviation for three samples of each ripeness, each consisting of a homogenate of three to five bananas, with ripeness assessed according to the criteria in Fig 1.

Fig 3 illustrates the fiber, starch, and sugar concentrations in the single lot of bananas assayed at the four levels of ripeness. Although there was a statistically significant difference (p<0.0001) in the moisture content between ripe and unripe bananas, the largest difference was 5.5 g/100g, between overripe (80.3 g/100g) and unripe (74.8 g/100g) and was not enough to meaningfully impact nutrient concentrations on a fresh weight basis. Therefore, component concentrations are presented on a fresh weight basis, being most relevant to fresh bananas consumed.

Dietary fiber measured by the EG method did not differ by ripeness. However, dietary fiber measured by the mEG method decreased going from unripe to overripe bananas, with a very large difference (14.0 g/100g) between unripe and slightly ripe and smaller changes (1.1–2.3 g/100g) among slightly ripe, ripe, and overripe. Starch followed a similar pattern, with a difference of 9.3 g/100g between unripe and slightly ripe and 1.4–3.0 g/100g among the slightly ripe, ripe, and overripe fruit. Within each ripeness stage, the content of glucose and fructose were similar, and there was no difference in content among slightly ripe, ripe, and overripe bananas, but a 5.0 g/100g difference compared to the unripe fruit. Sucrose was <1 g/100g in unripe fruit, increased to 2.5 g/100g in slightly ripe/ripe bananas, but in overripe fruit dropped to the same level as in unripe. Maltose was <0.5 g/100g in all samples.

The composition of the undried and dehydrated ripe bananas were compared on a dry weight basis. Total EG fiber, starch, fructose, glucose, and sucrose contents were unaffected by drying, but mEG total fiber was higher in dehydrated bananas (14.2 g/100g dry weight) compared to the undried bananas (11.6 g/100g dry weight). This analysis was done as an exploratory comparison, but illustrates the possible higher dietary fiber content of dehydrated bananas, presumably from creation of RS3 enzyme-resistant starch [32]. Formation of resistant starch can be affected by the drying method and conditions [32–34], so the dietary fiber content and carbohydrate composition bananas might vary depending on the production process.

#### Retail study

**Fig 4:**
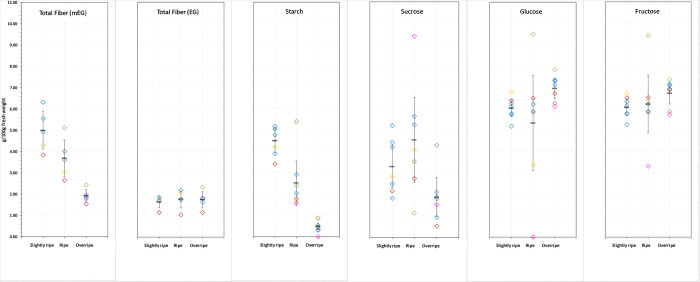
Dietary fiber, starch, and sugars in retail samples of bananas of the same ripeness (defined as in Fig 1). Data points in the same color are from the same sample (i.e., same hand(s) from the same lot, supplier, purchase point/time), where a portion of the bananas were allowed to ripen further, and the red symbols (◇) denote the ripening study samples. EG = enzymatic-gravimetric method (AOAC 991.43) [14]; mEG = modified enzymatic-gravimetric (“McCleary”) method (AOAC 2011.25) [16]. ─ mean, with error bars showing two times the standard error. Maltose was <0.5 g/100g in all samples.

Unlike in the ripening study, these samples represented different producers, supply chains, lots, post-harvest handling, ripeness at point of purchase, and size. As in the ripening study, there was a small but statistically significant difference (p<0.001) in the moisture content between overripe (78.6 g/100g) and slightly ripe and ripe bananas (75.4 g/100g), but the difference of 3.2 g/100g was not enough to meaningfully impact nutrient concentrations on a fresh weight/as-consumed basis and was similar to the moisture content at each ripeness stage in the ripening study (Fig 3). Table 2 summarizes the mean dietary fiber, starch, and sugars concentrations in bananas of each ripeness. Fig 4 illustrates the results for the individual samples relative to the mean values. Maltose was <0.5 g/100g in all samples.

**Table 2:** Mean and range for dietary fiber, sugars, starch, and proximate composition of retail samples of bananas of different ripeness (unripe bananas were not analyzed in retail samples).

Component (g/100g)	Slightly Ripe	Ripe	Overripe
**CARBOHYDRATES:**			
**Total dietary fiber, EG**	** **	** **	** **
Mean	1.64^A^	1.77^A^	1.75^A^
Range	1.15–1.86	1.05–2.20	1.14–2.33
*n*	*5*	*5*	*5*
**Total dietary fiber, mEG**	** **	** **	** **
Mean	4.99^A^	3.69^A^	1.93^B^
Range	3.84–6.32	2.66–5.12	1.55–2.44
*n*	*5*	*5*	*5*
**Starch**			
Mean	4.50^A^	2.52^B^	0.48^C^
Range	3.39–5.18	1.55–5.40	0.30–0.86
*n*	*7*	*7*	*6*
**Sucrose**	** **	** **	** **
Mean	3.33^A,B^	4.56^B^	1.88^A,C^
Range	1.84–5.25	1.15–9.40	0.53–4.31
*n*	*7*	*7*	*7*
**Fructose**	** **	** **	** **
Mean	6.08^A^	6.23^A^	6.72^A^
Range	5.26–6.72	3.33–9.43	5.73–7.38
*n*	*7*	*7*	*7*
**Glucose**			
Mean	6.03^A^	5.33^A^	6.96^A^
Range	5.18–6.78	<0.2–9.49	6.12–7.84
*n*	*7*	*7*	*7*
**Total sugars (glucose, fructose, sucrose)**			
Mean	15.0^A^	17.1^A^	16.7^A^
Range	12.9–16.6	14.4–19.4	14.2–19.6
*n*	*7*	*7*	*7*
**PROXIMATES:**			
**Moisture**	** **	** **	*** ***
Mean	75.57^A^	75.27^A^	78.58^B^
Range	74.97–77.02	72.15–76.92	75.87–80.56
*n*	*7*	*7*	*7*
**Ash**			
Mean	0.74^A^	0.66^B^	0.67^A,B^
Range	0.69–0.81	0.65–0.70	0.62–0.78
*n*	*6*	*6*	*6*
**Crude fat**			
Mean	0.37^A^	0.22^A^	0.22^A^
Range	0.10–0.72	<0.5–0.62	0.10–0.38
*n*	*6*	*6*	*6*
**Nitrogen**			
Mean	0.12^A^	0.12^A^	0.12^A^
Range	0.11–0.13	0.11–0.13	0.10–0.15
*n*	*6*	*6*	*6*

EG = enzymatic-gravimetric method (AOAC 991.43) [14]; mEG = modified enzymatic-gravimetric method. Different capital letter superscripts within each component indicate a statistically significant difference in means (p<0.05). Maltose was <0.5 g/100g in all samples.

For all components except EG fiber, there was wide variability in the concentrations among different samples at the same ripeness. As in the ripening study, there were no differences among ripeness in the dietary fiber measured by the EG method. In contrast to the controlled ripening study, there was no difference in mEG dietary fiber between slightly ripe and ripe bananas. Also, the difference in mean mEG dietary fiber between slightly ripe/ripe and overripe was ~1 g/100g greater. As in the ripening study, starch decreased going from slightly ripe to ripe and ripe to overripe, but the differences were about 1 g/100g larger. The mean concentrations of glucose and fructose at each ripeness stage (~6 g/100g each) and differences among stages of ripeness were similar to those in the ripening study. The mean sucrose content of the retail banana samples did not differ between slightly ripe and ripe bananas and the content in overripe bananas was lower, as seen the ripening study, but the absolute sucrose contents were higher, by ~ 1–1.5 g/100g.

Some differences in composition due to size and other criteria that were controlled in the ripening study, but not in selection of retail samples, may have contributed to overall variability among retail samples. For example, in the ripening study, in which the ripe and unripe medium and extra-large size bananas were analyzed, a small but statistically significant (p<0.05) difference was found between sizes for some components. These components and concentrations in g/100g fresh weight were, for medium and extra-large, respectively: EG total fiber (1.23, 0.88) and moisture (76.42, 77.41) in ripe bananas; mEG fiber (17.9, 16.0), fructose (1.49, 1.69), glucose (1.55, 1.75), and moisture (74.78, 75.88) in unripe bananas. Obviously other variables among samples contribute to the larger variability seen among retail samples at the same assessed ripeness.

## Discussion

### Carbohydrate content and composition

Carbohydrates comprise approximately 70–80% of the dry mass of the unripe banana, consisting of starch (primarily) and non-starch polysaccharides (NSP) (for example, hemicellulose, pectin, cellulose), as well as smaller amounts of fragments of the polysaccharides formed during cell wall breakdown and starch catabolism during ripening [13,35]. The respiration rate is low in green bananas, and ripening begins at the climacteric, instigated by the production of ethylene and accompanied by a rapid increase in respiration [36]. During ripening starch is converted to sugars, resulting in the softening of texture and sweet taste associated with the ripe banana. These processes involve numerous hydrolases acting on starch and NSP, including sucrose synthase and invertase, among others [35,37]. Bananas are harvested at different stages of ripeness, but primarily unripe (green) when that fruit will be transported outside the growing region, as is the case for bananas in retail markets in the U.S. Various methods and treatments are used to control post-harvest ripening and prevent spoilage in bananas packaged for transport [36,38,39].

The most striking finding in this study, which looked at the composition of bananas from a macronutrient perspective, was the higher dietary fiber content in unripe and ripe bananas when measured by the currently accepted modified enzymatic-gravimetric method (AOAC 2011.25) [16] compared to the longstanding standard enzymatic-gravimetric method (AOAC 993.41) [14], which decreased with increasing ripeness. Dietary fiber measured by the mEG method exceeded the amount determined by the EG method by an average of 15.6 g/100g in unripe bananas (ripening study) to 3.4 and 1.9 g/100g in slightly ripe and ripe bananas, with no difference between mEG and EG fiber in overripe bananas. Given the high starch content of bananas, which decreases during ripening, this finding is consistent with the quantitation of resistant starch (RS) by the mEG (but not EG) method. Bananas have been reported to contain a significant amount of RS [12], with levels decreasing throughout ripening, and RS is considered dietary fiber according to the current definition [9].

Perhaps the most touted aspect of the mEG compared to the EG method is that it includes non-digestible low molecular weight oligosaccharides (LMWSDF) (for example, fructans, stachyose, raffinose) present in some foods such as legumes and some fruits [40,41]. However, another difference is that the mEG method does not involve initial “cooking” (autoclaving or boiling) that renders some types of resistant starch accessible to digestive enzymes. There are five types of RS, and bananas contain RS2 (granular, native starch with a highly crystalline structure [32]). LMWSDF was not detected at >0.8 g/100g in any of the banana samples in this study, but the impact of the enzymatic hydrolysis conditions was notable. Our previous work showed that the underestimation of fiber by the EG method can be significant, particularly in high starch foods [42], and this study demonstrates that those differences deserve more consideration in distinguishing the dietary fiber content of high starch foods consumed at different stages of ripeness or maturity or prepared in different ways, which can have varying effects on starch and different types of resistant starch.

Moreover, the production of limit dextrins and pectic and hemicellulosic oligosaccharides in ripening bananas, which are likely at levels below the limit of detection for LMWSDF in the mEG assay, should be considered. If all of the difference in fiber quantified by the mEG and EG methods was due to resistant starch, the decrease in starch and mEG fiber would be the same. Among the slightly ripe, ripe, and overripe bananas, there was no detectable difference in the amount by which mEG fiber and starch decreased from slightly ripe to ripe and from ripe to overripe (2 g/100g). However, going from unripe to slightly ripe, the difference between mEG and EG fiber exceeded the decrease in starch by 4 g/100g. Better methods to measure the concentrations, composition, and structure of the carbohydrates comprising “dietary fiber” are important given the contemporary focus on the effects of diet on the gut microbiome and its association with health outcomes [43–45].

### Sugars

In bananas, sucrose is derived from the breakdown of starch into maltose and glucose, which are then converted to sucrose through the action of sucrose synthase, and sucrose is then further broken down into glucose and fructose by invertase as the fruit ripens [46–48]. In this study, the predominant sugars at any ripeness stage were glucose and fructose (in approximately equal amount at all ripeness levels) at, on average 12–13 g/100g total in slightly ripe to overripe and 3.2 g/100g in unripe bananas. Sucrose increased only nominally going from unripe (~1 g/100g) to ripe (4.6 g/100g), with only a 1–2 g/100g difference between slightly ripe/ripe and ripe/overripe. No maltose was detected (<0.15 g/100g) in any sample, of any ripeness. On average, sucrose comprised only 25% of the sugars in unripe bananas, 22–27% in slightly ripe/ripe bananas and 11% in overripe bananas, out of total sugar contents of 4.3 and 15–17 g/100g in unripe and slightly ripe to overripe bananas, respectively.

The results for soluble sugars (fructose, glucose, sucrose, and maltose) in some reported ripening studies differed both from each other and from those obtained in the current study [49–51]. Contrary to the results reported here, sucrose has been previously observed to be the most abundant soluble sugar derived from starch catabolism during banana ripening in several cultivars, including Cavendish [46,52,53]. However, there have also been several studies in which fructose and glucose were found to be the most abundant soluble sugars present in ripened bananas [49–51]. Moreover, maltose has also been detected in a wide range of concentrations in ripening bananas [49,50], while none was detected in the present study. The processes governing the catabolism of starch into soluble sugars is complex and may be influenced by several factors including growth conditions, point of origin, ripening conditions, and post-harvest storage and treatment [49,53,54].

Also worth noting is that in the present study, whereas the changes in total mEG fiber and starch were in the same direction between stages of ripeness in particular individual samples that were assayed at more than one ripeness compared to the trend in the overall mean, the same was not true for the sugars, where individual samples in some cases showed a large difference in the direction opposite of the mean differences by ripeness (see Fig 4). Our hypothesis is that cultivar, growing conditions and post-harvest handling and treatments likely affected sugar metabolism. The specific cultivar, country of origin, growing conditions and post-harvest treatment were not controlled variables in this study. The samples reflect typical U.S. marketplace selection, including in some cases bananas allowed to ripen after purchase, and represented major producers in the U.S. market. While all were *Musa* spp. (AAA Group, Cavendish Subgroup), different cultivars are widely distributed in the U.S. retail market, including Dwarf Cavendish, Grade Nain, and Giant Cavendish [6]. It has been shown that starch breakdown and sucrose synthesis in different banana cultivars are affected differently by the exogenous application of ethylene [55,56], with Cavendish responding with earlier ripening, starch degradation, and sucrose production, and these processes are mitigated by potassium permanganate [57]. Potassium permanganate has been used to delay post-harvest ripening of bananas [58] and represents one of a variety of post-harvest treatments used to control ripening and fruit quality [39,59]. Starch breakdown and sugar synthesis and metabolism is a complex process influenced not only by ethylene but other hormones, the control and interaction of which are not fully understood [46]. Growing conditions have also been shown to affect the composition of the fruit [60,61]. Thus, it is not unreasonable to presume that differences in ripening conditions, treatments used to control ripening, cultivar, transport times, and other variables influence the ultimate content and relative concentrations of sugars and starch in bananas at the point of consumption. The sample-to-sample variability in sugar content and composition in this study support the influence of post-harvest factors in sugar metabolism in the ripening banana, and further exemplify why food composition databases should reflect these factors when reporting the nutritional content of fruits and vegetables as consumed.

### Analytical methods

The impact of analytical methods on values should be considered, not only for fiber, but also for sugars and starch. The carbohydrate components expected in banana fruit include starch, non-starch polysaccharides (NSP) (including hemicellulose, pectin, cellulose), malto-oligosaccharides, limit dextrins, pectin and hemicellulose fragments, monosaccharides and disaccharides. The maltooligosaccharides, limit dextrins, and pectin and hemicellulose fragments are not accounted for in the routine analysis of sugars (defined as mono- and disaccharides) or by the mEG fiber or starch methods. Larger NSP fragments would precipitate as soluble fiber and be included in fiber recovered in the gravimetric portion of the mEG method. However, smaller limit dextrins and some oligosaccharide fragments of the NSP could be missed in the liquid chromatographic portion of the assay [62], due to either being below the limit of detection or not being specifically identified and quantified. Extractability of NSP directly from banana pulp has also been shown to be affected by the soluble sugars content and method of extraction [63], as has the recovery of sugars and oligosaccharides [50]. It is also possible that depending on where the sugars and starch are compartmentalized in the ripening banana that the standard method of sugar extraction used in this work did not fully release all sugars from the matrix, in which sucrose is synthesized from glucose and maltose released from starch, within cell plastids [46]. In fact, no maltose (<0.1 g/100g) was found in any sample at any ripeness stage in this study, yet it would be expected to be found as a breakdown product from starch in ripening bananas. In our study, all samples were kept frozen prior to analysis, which is important to prevent potential loss of sucrose, which has been shown to invert to glucose and fructose in some foods stored at refrigeration temperatures [64], and it is important to pay attention to sample preparation and handling when evaluating data reported for sugars in foods.

The effect of freezing itself does need to be considered in the fiber analysis. Slow cooling and freezing result in retrogradation and production of RS3 resistant starch in cooked high-starch foods [65]. The effect of freezing, and particularly flash freezing in liquid nitrogen (as used in homogenization of samples in this study) on the digestibility of starch in the in vitro mEG assay remains to be explored. Technically the mEG procedure allows freeze-drying samples (which was not done in the present study). However, freeze-drying, in addition to heating, can affect the assayed dietary fiber content in some foods [66] and specifically affects banana starch [67]. Thus, freeze-drying should be specifically prohibited in any enzymatic method attempting to mimic digestion.

Ideally there would be a more direct and comprehensive method for quantifying all individual non-digestible, prebiotic carbohydrates comprising the totality of carbohydrates in a food, using methods with an adequate limit of detection and in which sample preparation and analysis do not involve conditions that alter the food as consumed. Notably, it is possible that particular components have a prebiotic effect at levels that are too low to detect when analyzed in the LMWSDF fraction left over from the mEG fiber assay, in which sample weights and solvent volumes have been optimized primarily for the gravimetric portion of the assay. Newer, high-throughput mass spectrometric methods that quantify carbohydrates and examine linkages [68,69] will be useful in this effort.

### Implications for food composition data

#### Dietary fiber

The impact of changes in the definition of dietary fiber and analytical methodology on food composition data have been discussed previously, especially for foods containing significant amounts of starch that are consumed uncooked/unprocessed [42]. For bananas, the mean total dietary fiber content previously reported in the USDA Legacy SR database (determined by the traditional EG assay) is 3.1 g per 118g medium size banana [7], NDB# 009040), with no distinction among bananas of different ripeness. The present study found a mean dietary fiber content of 2.2, 4.4 and 5.9 grams per medium banana for overripe, ripe and slightly ripe, respectively, when determined by the mEG method. In contrast, the mean dietary fiber content of bananas at all stages of ripeness was similar to the value in the Legacy SR database when measured by the EG method (2.0 g/118g banana). Many other foods contain resistant starch [70] and could be expected to have dietary fiber contents that would be impacted by method of analysis. In fact, decades ago, it was reported that bananas contain a large portion of non-digestible starch that decreases with ripening [71], yet determination of dietary fiber in foods (including in the USDA Legacy SR food composition database) has largely been determined by the EG approach.

The physiological effects and health outcomes related to dietary fiber intake and different types of dietary fiber have been the subject of extensive research for more than 50 years [72,73]. In the U.S., the current Dietary Reference Intake (DRI) for total fiber is 14 g/1000 kcal [74]. However, there is no DRI for different components of “dietary fiber”. Historically, fiber was first distinguished as water-soluble and insoluble, to which different benefits were attributed, including cholesterol sequestering for soluble fiber and decreased intestinal transit time for insoluble fiber [75]. More recent investigations have explored how individual components included in dietary fiber exert their physiological effects through interaction with the gut microbiome [76–78]. These interactions and effects have been shown to be structure specific [79]. Thus, it has become increasingly necessary to understand dietary fiber in greater detail. Bananas are known to contain both oligo- and polysaccharide structures that contribute to dietary fiber such as fructans, resistant starch, cellulose, xyloglucans, mannans, and pectin [80,81]. How these fiber components are affected by fruit ripening, point of origin, or post-harvest treatment is yet to be fully understood.

In this study, dietary fiber measured by the mEG method versus the EG method was higher by up to ~3 g per medium banana, which is ~20% of the 14g/1000 kcal DRI. For bananas it is therefore recommended that enzymatic-gravimetric dietary fiber be quantified using the mEG method, which provides a better measure of the total non-digestible carbohydrates to which the gut microbiota is exposed. Attention should also be paid to differences in dietary fiber content of bananas as a function of ripeness, especially in the underripe or slightly ripe fruit. The effect of dehydration on dietary fiber content of bananas also suggests caution in imputing the dietary fiber content of dried fruits from values for fresh fruits. An actual difference in dietary fiber content and composition may be a factor in health claims for dried versus fresh fruits [82] and should also be considered in assessing intake of dietary fiber and the physiological impact of specific dietary fiber components and foods. The potential health effects of RS have been reported in human studies, most notably in relation to managing diabetes and an approved European Union health claim [65,83]. Reported effects of RS on gut health have led to its consideration as a prebiotic [84] and food manufacturers have included it as an ingredient in some foods [85]. The present work suggests that the changes in mEG fiber may reflect changes in RS, which may impact physiological responses to the consumption of bananas and add to the complexity of dietary recommendations. There is also a fraction of carbohydrates likely unaccounted for by the mEG method, which varies with banana ripeness, that includes the non-digestible oligosaccharides resulting from starch hydrolysis [86].

#### Calculated energy

**Fig 5:**
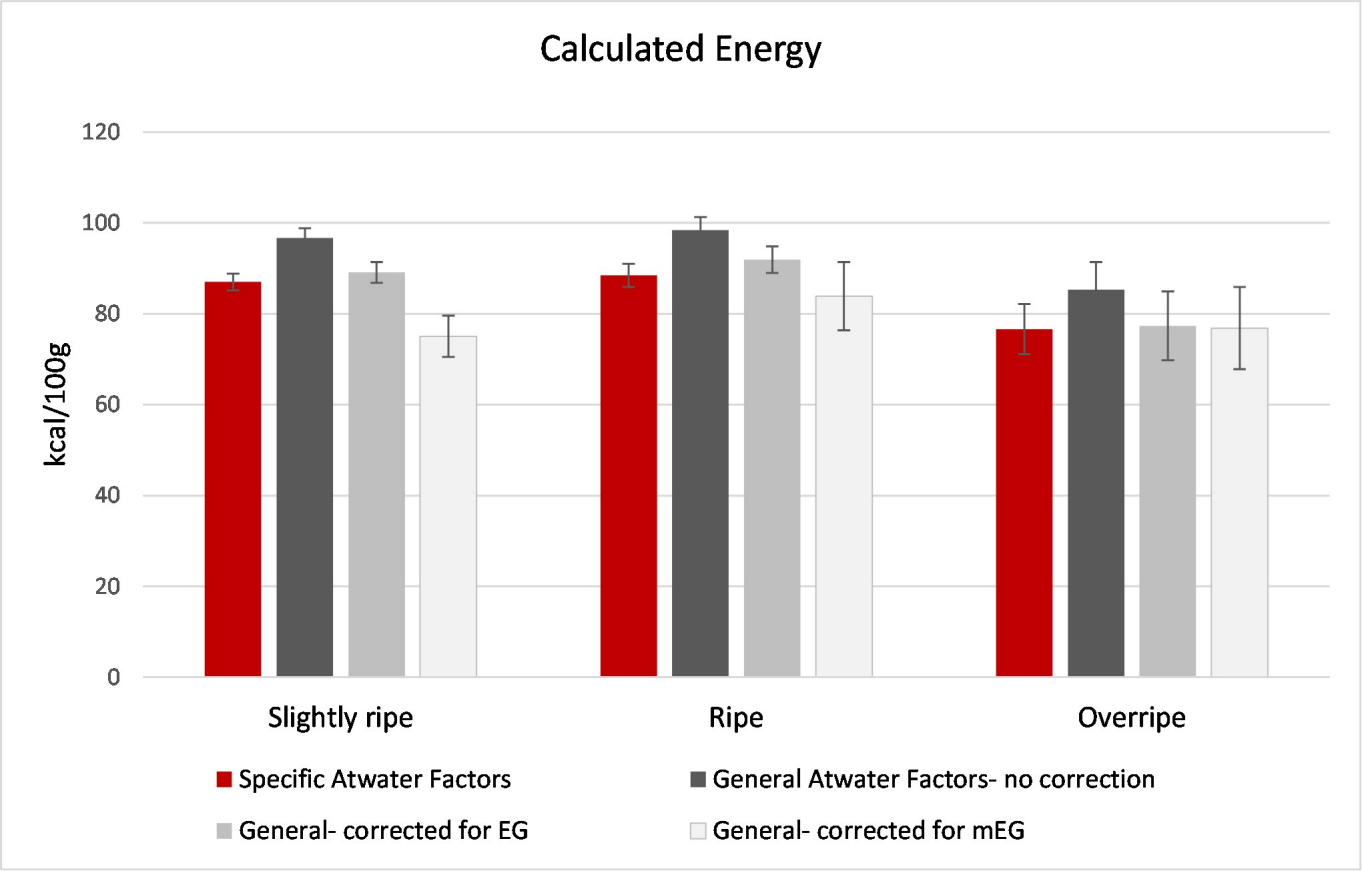
Mean energy content of bananas of different ripeness in the retail study, calculated from proximate data (Table 2) using either the specific or general Atwater factor for carbohydrates by difference (3.6 and 4 and kcal/g, respectively) [87], with and without subtracting EG fiber or mEG fiber (see Table 2) from total carbohydrates by difference before applying the general factor. Error bars show the range for 6 samples at each stage of ripeness.

Energy (kcal per 118g medium size banana) was calculated from assayed proximates (Table 2) using specific Atwater factors of 8.37, 3.36, and 3.6 kcal/g for fat, protein (N*6.25) and carbohydrate by difference [87] and compared to energy calculated using the general Atwater factors of 9, 4, and 4 kcal/g for fat, protein, and carbohydrate by difference with and without mEG or EG fiber subtracted (“adjusted”). Because proximates were not measured in the ripening study, only data for slightly ripe, ripe, and overripe bananas from the retail study samples could be compared. The mean specific and mean adjusted energy content did not differ for ripe and overripe bananas and was only 13 kcal per banana different for the slightly ripe (with values derived from specific Atwater factors being higher). Because the specific Atwater factors are empirically determined based on metabolizable energy in the particular food [87], and nearly all energy from bananas is derived from carbohydrates, this agreement is expected if non-digestible fiber (mEG) is subtracted from carbohydrate by difference and the non-specific Atwater factor is applied (Fig 5).

Proximate components were not measured in the ripening study, and only the ripening study involved unripe bananas. However, given the large difference in mEG fiber between unripe and slightly ripe (16.5 g/118g medium banana) and small differences among subsequent ripeness levels (< 3 g/banana), and the fact that there was a small and statistically significant (p<0.05) decrease in energy calculated with the different energy factors for slightly ripe versus ripe bananas, it is reasonable to assume that there would be an even larger difference in the energy content of unripe bananas calculated using the specific Atwater factors. For example, assuming 16.5 g mEG fiber per medium banana and 30 g total carbohydrate by difference, 0.3 g fat, and 0.8 g protein, the specific and adjusted calculated energy contents are 114 and 61 kcal per medium banana, respectively. Any application using unripe bananas (not further cooked) would likely have the energy contribution from the bananas overestimated significantly if the specific Atwater factors for bananas were used for the calculation, as a result of the high content of non-digestible starch. More importantly than absolute energy values, much of the clinical concern about different foods, in particular those high in carbohydrates, involves the glucose/insulin response and what the microbiota are exposed to in the gut. Other fruits and vegetables that are high in starch or fiber are consumed from the unripe to the very ripe stage, including plantains (another group of cultivars of the *Musa* genus that are widely consumed throughout the world) and experience less starch degradation during ripening [88].

## Conclusions

From an epidemiological perspective, there is a need for more detailed food composition data, including carbohydrate composition, among fruits and vegetables at different stages of maturity and ripeness. This study demonstrated that the dietary fiber, sugar, and starch content of bananas cannot be generalized across ripeness stages, and food composition databases typically do not distinguish between bananas at different stages of edible ripeness. Some of the carbohydrate differences detected in a single lot of bananas analyzed at the slightly ripe versus ripe stage were non-existent in retail samples. Therefore, caution should be exercised in extrapolating results from controlled studies or field studies to the composition of the food as consumed, given myriad other variables in the supply chain. Large differences in dietary fiber measured by the traditional (EG) and more recently modified (mEG) approaches also suggest that more specific, sensitive, and economical methods are needed to characterize and quantify the actual composition of the non-digestible carbohydrates comprising “fiber” and the changes that occur during ripening. As more and more research demonstrates the role of the gut microbiota in health and disease, epidemiologists, nutritionists, and food analysts must move away from the focus on the “dietary fiber” (i.e. non-digestible residue) of foods and towards considering individual components and their effects on the gut microflora. Other fruits and vegetables that are high in starch or fiber and consumed from the unripe to very ripe stage, including plantains (cultivars of the *Musa* genus that are widely consumed throughout the world), are worthy of more detailed analysis of the carbohydrate content and composition and variability in the food as consumed.

## Supporting information

S1 Table Data for individual samples. Results for analyzed components in individual banana samples.
(XLSX)
